# The Role of Methionine in the Formation of Key Aroma Compounds in Microwaved Walnuts

**DOI:** 10.3390/foods15040719

**Published:** 2026-02-15

**Authors:** Yishen Cheng, Yilang Liu, Haonan Zheng, Kexi Ma, Jiachen Zang, Lei Zhang

**Affiliations:** 1 College of Food Science and Nutritional Engineering, China Agricultural University, Beijing 100083, China; yishencheng@cau.edu.cn (Y.C.); areyoucat@cau.edu.cn (Y.L.); zhhn_2003@163.com (H.Z.); makexi2009@163.com (K.M.); zangjiachen@cau.edu.cn (J.Z.); 2 College of Food Science and Pharmacy, Xinjiang Agricultural University, Urumqi 830052, China; 3 Xinjiang Walnut Processing Engineering Technology Research Center, Kashgar 844000, China

**Keywords:** walnut aroma formation, microwave treatment, methionine, key aroma compounds, GC-MS analysis, flavor enhancement

## Abstract

Given the growing consumer preference for plant-based proteins, improving their flavor profiles is essential for market success. Despite their nutritional benefits, plant proteins, such as walnut proteins, often suffer from weaker and less appealing flavors than those of animal proteins. This study investigated the pivotal role of amino acids in walnut aroma development during microwave treatment. Through gas chromatography mass spectrometry analysis of five walnut varieties, considerable differences in the aroma compound composition were identified with 13 key aroma components highlighted via relative odor activity value analysis. The present results demonstrated that methionine played a predominant role in pyrazine and heterocyclic compound production, underscoring its importance in walnut aroma formation. Thus, heat treatments, particularly microwave processing, show potential for enhancing flavor under the conditions investigated. These findings suggest a possible approach for improving flavor profiles in plant protein–based systems.

## 1. Introduction

As consumer health demands have increased, plant proteins have gradually received more attention. Both health and sensory attributes strongly influence consumer acceptance [1]. However, many plant protein ingredients are associated with characteristic flavor challenges, often arising from non-protein components or processing-related reactions, which may result in undesirable or off-flavors. These sensory limitations can negatively affect consumer acceptance of plant protein–based products [2]. Therefore, improving flavor quality is essential for the further development and broader application of plant protein products.

Walnut is particularly notable for its nutritional richness and popularity. Walnuts (*Juglans regia* L.) are a plant species in the walnut family (*Juglandaceae*) and are one of the most widely distributed tree nuts owing to their high nutritional value [3]. The U.S. Food and Drug Administration has endorsed walnuts as a dietary supplement with various health benefits, including the potential to lower low-density lipoprotein levels and reduce the risk of coronary heart disease [4]. This endorsement has led to the incorporation of walnuts in many food products aimed at health-conscious consumers. Walnuts have an oil content of up to 62–68% and contain a high amount of polyunsaturated fatty acids (PUFA), especially omega-3:omega-6 PUFAs, and an optimal balance of micronutrient content [5]. Therefore, walnut oil is an important component of deep walnut processing and has good prospects. In addition, heat pre-treatment of walnuts (e.g., roasting, microwave, and radiofrequency) can greatly improve walnut oil flavor. Typically, heat pre-treatment of walnuts leads to characteristic nutty and roasted flavors, which can be attributed to the presence of pyrazine compounds [6].

The effects of heat treatment on walnut flavor profiles have been demonstrated. Aroma compounds, such as aldehydes, furans, and pyrazines, have been detected during the roasting of walnuts, with furfural being one of the most abundant compounds [7]. Similarly, a considerable increase in the content of seven pyrazines in walnut oil after microwave treatment contributed to a distinctive roasted flavor [8]. Furthermore, Zhou showed that roasting led to a rapid increase in pyrazines, a robust toasted flavor in walnuts, thus underscoring the critical role of heat treatment in defining walnut flavor profiles [9].

Due to its energy-saving characteristics and rapid heating capability, microwave treatment is increasingly being employed as a pretreatment method for extracting oil from nuts. Compared with conventional heating methods, microwave processing can markedly influence the volatilization rates and relative proportions of aroma-active compounds, thereby contributing to the development of more intense roasted and nutty flavors [10]. However, rapid volumetric heating and high energy input over short durations also affect other key quality-influencing components. Microwave treatment may reduce the content of certain heat-sensitive or unstable compounds (e.g., phenolic constituents) while potentially triggering transformations among phenolic compounds [11]. In addition, microwave heating has been reported to influence the stability and antioxidant properties of nuts, depending on processing conditions [12,13]. Overall, microwave treatment can be regarded as a relatively rapid and efficient heating approach that promotes aroma development in nuts.

Microwave treatment plays a key role in improving walnut flavor mainly by promoting lipid oxidation and Maillard reactions. Linoleic acid and other lipid precursors in these reactions promote the formation of unstable dihydropyrazine compounds through aldehydes during lipid degradation. This ultimately leads to the synthesis of long-chain pyrazines, which are closely related to the baking aroma of walnuts [14]. In addition, the amino acids in walnuts play an important role in the Maillard reaction by interacting with reducing sugars to ultimately produce a variety of heterocyclic compounds, such as pyrazines, pyridines, and pyrroles, with flavor profiles. The Maillard reaction is a non-enzymatic browning reaction that occurs during food processing and is associated with the production of various flavor and volatile compounds. This reaction involves the loss of water molecules between the amine group of an amino acid and the carbonyl group of an aldehyde or ketone of a reducing sugar [15]. In the Maillard reaction, pyrazines are firmly associated with the flavor and aroma of roasting. Pyrazine compounds are formed by the condensation of two amino carbonyl groups produced by Strecker degradation during peanut roasting [16]. At this stage, different amino acid precursors substantially influence the formation of flavor substances [17]. Zhang confirmed that the addition of amino acids, such as lysine and arginine, promotes the formation of pyrazine flavor compounds in the Maillard reaction system composed of amino acids and sugars [13].

Therefore, it is essential to clarify how amino acid composition influences aroma compound formation during microwave processing. Changes in the amino acid composition of raw walnut materials have an important effect on the flavor of walnut oil. Hence, this factor can be potentially applied in industrial production to influence flavor. However, direct evidence linking specific amino acids to key aroma compounds have been largely unexplored prior to this study. Therefore, the correlation between the amino acid composition and aroma components in different varieties of raw walnut materials was carried out in this study.

## 2. Materials and Methods

### 2.1. Walnut Samples and Microwave Treatment

As shown in Table 1, Five distinct walnut types were collected from the Xinjiang Uygur Autonomous Region, China, where walnuts are widely cultivated. All samples were harvested in the same year as the experiment and transported to the laboratory under refrigerated conditions shortly after harvest. After collection, the husks were carefully removed manually, and the walnuts were stored at 4 °C in a cold room prior to analysis to minimize quality deterioration.

Before microwave treatment, the walnuts from each location were numbered and manually shelled to remove the walnut shells and septa. 500 g of peeled walnut kernels were evenly spread in a single layer on a tray, with an approximate thickness of 8 cm. The samples were then subjected to microwave heating at 800 W for 4 min using a microwave oven (model MWBLXE1ACM, Wuhan, China). After microwave heating, the walnuts were ground into powder using a high-speed pulverizer (model ZG-0317, Jinhua, China) operating at 36,000 r/min, and the powder was passed through a 300-mesh sieve to obtain a uniform particle size. The resulting walnut powder was immediately placed into self-sealing bags for subsequent analyses.

### 2.2. Headspace Solid-Phase Micro-Extraction (HS-SPME)

The microwave-treated walnut kernel samples were pulverized, and the walnut powder was placed in a self-sealing bag for use. Crushed and homogeneous walnut kernel (2.0 g) was placed in a 20 mL HS-SPME sample bottle, and 5 μL of o-dichlorobenzene-dichloromethane solution (0.0325 mg/mL) was added as an internal standard and a magnetic rotor was added. A magnetic stir bar was then placed in the vial, which was tightly sealed with a PTFE/silicone septum cap. After equilibrating at 60 °C for 20 min on a magnetic stirrer at a constant stirring speed of 300 rpm. Subsequently, an aged divinylbenzene/carboxen/polydimethylsiloxane (DVB/CAR/PDMS) fiber with a length of 2 cm and a thickness of 50/30 μm was used to extract the sample. After aging, the DVB/CAR/PDMS extraction head (length = 2 cm and thickness = 50/30 μm) was inserted into the headspace vial at 1 cm above the walnut kernel and adsorbed at 60 °C for 30 min. Thereafter, the HS-SPME extraction head was pulled out, inserted into the gas chromatography mass spectrometry analysis (GC-MS) inlet, and thermally desorbed at 250 °C for 8 min.

### 2.3. Gas Chromatography Mass Spectrometry Analysis

The GC-MS conditions were as follows: HP-5MS capillary column (30 m × 0.25 mm, 0.25 μm), and the carrier gas was high-purity helium (>99.999%) at a flow rate of 1 mL/min. Solid-phase microextraction was performed with manual injection without split injection, the temperature of the injection port was 250 °C, and the thermal desorption time was 8 min. The temperature increase program was as follows: held at 50 °C for 2 min, increased at 5 °C/min to 200 °C, further increased at 5 °C/min to 230 °C, and held for 5 min. The mass spectrometer was operated using an electron impact (EI) ionization source, with the ion source temperature set at 230 °C and an electron energy of 70 eV. The quadrupole temperature was maintained at 150 °C. Data were acquired in full-scan mode over a mass range of 50–350 *m*/*z*.

### 2.4. Identification, Quantification and Relative Odor Activity Value (ROAV) Analysis of Volatile Compounds

The GC-MS experimental data were preliminarily characterized using similarity searches of the NIST17 database and further characterized by combining them with the linear retention indices of the corresponding compounds in the references. The n-alkane (C7–C40) standards were analyzed under the same GC-MS conditions for direct liquid uptake. Thereafter, the linear retention indices were calculated, and the volatile compounds in the samples were further characterized by comparing the retention indices with those of the corresponding substances in the references of the NIST17 database. the linear retention index was calculated according to Formula (1):

(1) $$\mathrm{The}\;\mathrm{linear}\;\mathrm{retention}\;\mathrm{index}\;=\;100\times (n+\frac{t_{x}-t_{n}}{t_{\left(n+1\right)-t_{n}}})$$

Where $t_{x}$, $t_{n}$x, $t_{n}$, and $t_{\left(n+1\right)}$ are the retention times per minute for the substances to be measured x, with n, (n + 1) carbon atom n-alkanes, respectively.

Volatile compounds were quantified using the internal standard method

ROAV was calculated using the method of Zhu [18] as follows:

(2) $${OAV}_{i}=\frac{C_{i}}{{OT}_{i}}$$

(3) $${ROAV}_{i}=100\times \frac{{OAV}_{i}}{{OAV}_{max}}$$

where ${OAV}_{i}$ represents the odor activity value, ${ROAV}_{i}$ represents the relative odor activity value, and ${OAV}_{max}$ is the maximum odor activity value. The relative concentration ($C_{i}$) was determined via GC-MS. The odor threshold values (${OT}_{i}$) for the compounds in solid media were sourced from the relevant literature.

### 2.5. Amino Acid Profile Analysis

Free amino acids (FAA) were determined using a ninhydrin-based method with an automatic amino acid analyzer. FAA were extracted from raw and microwave-treated walnut kernels with 80% (*v*/*v*) ethanol and reconstituted in citrate buffer (pH 2.2). Norleucine was used as an internal standard. FAA analysis was performed using a Biochrom^®^ 20 plus amino acid analyzer (Harvard Bioscience, Holliston, MA, USA) following the manufacturer’s standard procedure, with post-column ninhydrin derivatization and detection at 570 nm and 440 nm. Amino acid concentrations were quantified using an external standard method. All analyses were conducted in triplicate.

### 2.6. Preparation of Maillard Reaction Products Derived from Amino Acids and Glucose

To investigate the influence of amino acids on the Maillard reaction, a simplified amino acid–reducing sugar model system was employed. Aqueous solutions of individual amino acids—methionine (Met), proline (Pro), or glutamic acid (Glu)—and glucose were prepared at a concentration of 0.2 mol/L each. The amino acid and glucose solutions were mixed at a 1:1 molar ratio, and the total reaction volume was adjusted accordingly. The initial pH of the reaction mixture was adjusted to 8.0 ± 0.1 using 6 mol/L NaOH. Aliquots of the reaction mixtures were transferred into glass vessels, which were loosely capped to avoid pressure buildup, and heated in a microwave reactor at 800 W for 4 min. Reactions were conducted under ambient atmospheric conditions. After heating, the reaction vials were immediately cooled to room temperature. The reaction products were then either used directly for subsequent analysis or stored at 4 °C prior to analysis.

### 2.7. Statistical Analysis

Analysis of variance was performed using Duncan’s test (*p* > 0.05). Box plots were constructed using GraphPad Prism version 9. Origin 2022 was used for the statistical analysis and graphing of the principal component analysis, Venn diagrams, and radar plots. Partial least squares regression (PLSR) analysis was performed using XLSTAT version 2019 to illustrate the correlations between odor-active compounds and amino acids.

## 3. Results and Discussion

### 3.1. Volatile Compounds of Five Walnut Cultivars

The aroma profiles of fresh walnuts from different cultivars are presented in Figure S1 (Supplementary Material). In untreated walnuts, the volatile profile was mainly composed of aldehydes, alcohols, and organic acids, such as hexanal, 1-hexanol, 1-pentanol, and hexanoic acid, which are commonly associated with lipid oxidation and endogenous metabolism in fresh nuts. Notably, heterocyclic compounds, including pyrazines and furans, were absent or detected only at trace levels in untreated samples.

However, a total of 32 volatile compounds were detected in the five walnut cultivars after microwave heating. During the heating process, walnuts underwent varying degrees of lipid oxidation and Maillard reactions, resulting in the formation of volatile compounds that contribute to walnut flavor. The identified volatile compounds included thirteen heterocyclic compounds, six alcohols, three acids, nine aldehydes, one ketone and one other compound. Heterocyclic compounds were the most prominent components of walnut volatile compounds after microwave treatment. Aldehydes were the second most abundant volatile compounds, followed by alcohols.

The quantitative data of the identified volatile compounds are summarized in Table 2. It should be noted that relatively large standard deviations were observed for some volatile compounds. This variability is likely related to the intrinsic characteristics of HS-SPME extraction, which is sensitive to matrix effects, as well as the high lipid content of walnuts. Although standardized grinding and sieving procedures were applied, slight heterogeneity in particle size and oil distribution may have influenced the adsorption behavior of volatile compounds. Nevertheless, all samples were analyzed under identical conditions with replicate measurements, and statistical analysis was applied; therefore, the observed variability does not affect the overall trends or conclusions.

**Figure 1:**
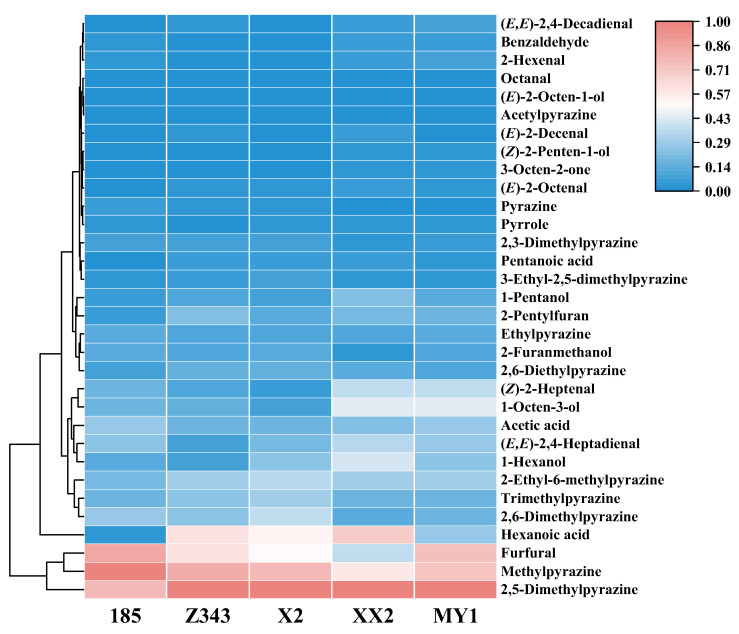
Heatmap of volatile compounds in five walnut cultivars after microwave treatment.

A heat map presents a large amount of data in a more intuitive matrix block clustering to analyze the differences, similarities, and correlations between variables and to better reveal the changes and patterns of the samples. The differences in the composition and content of volatile compounds after microwaving different varieties of walnuts are shown in Figure 1. The red color indicates a high content, darker red color indicates a higher content of the compound, blue color indicates a low content, and darker blue color indicates a lower content of the compound. As shown in Figure 1, cultivar-dependent differences in volatile profiles were observed among the five walnut cultivars after microwave treatment. Furfural exhibited pronounced variation, with higher levels in cultivars 185 and Z343, whereas cultivar XX2 consistently showed the lowest content. Similar trends were observed for hexanoic acid and 1-octen-3-ol, both of which were more abundant in cultivars 185 and Z343 but markedly lower in XX2.

Pyrazine compounds, which are characteristic products of Maillard reactions, also displayed cultivar-specific differences. Although pyrazines were detected in all cultivars, XX2 generally exhibited lower levels of several key pyrazines, such as methylpyrazine and 2,5-dimethylpyrazine, whereas 185 and Z343 tended to show higher abundances. Notably, the extent of variation differed among individual pyrazines, suggesting compound-specific cultivar effects.

The dominance of pyrazines and furans observed in the present study is in good agreement with previous reports on microwave-treated walnuts [19]. Zhou et al. reported that microwave pretreatment enhanced roasted flavor attributes in walnuts, accompanied by an increased formation of pyrazine compounds and typical color changes [14]. Similarly, Wu et al. identified methylpyrazine and 2-ethyl-5-methylpyrazine as key contributors to the flavor of microwave-treated walnut samples compared with other heating methods [20].

Taken together, these cultivar-dependent differences in specific volatile compounds indicate that precursor availability varies among walnut cultivars. Differences in amino acid and reducing sugar composition may therefore influence the extent of Maillard reactions. Under microwave heating conditions, this variation is likely to contribute to the observed differences in volatile compound formation.

### 3.2. Quantification of ROAV and Evaluation of Odor Characteristics

As shown in Table 3*,* ROAV is a metric that takes into account the interactions between the odor compounds and the food matrix while also representing the contribution of each compound to the overall aroma. ROAV is the ratio of the relative concentration of each compound to its absolute threshold, and compounds with ROAV > 1 are considered to be the major odor compounds [21]. As shown in Table 2, 13 aroma compounds with ROAV > 1 were calculated by the formula in the microwaved walnut samples.

Among the identified compounds, 2-ethyl-6-methylpyrazine exhibited the highest ROAVs across most walnut cultivars. Its high ROAV can be attributed to a low odor threshold and a strong roasted and nutty aroma. Other pyrazines, including methylpyrazine, trimethylpyrazine, 2,5-dimethylpyrazine, 3-ethyl-2,5-dimethylpyrazine, 2,6-diethylpyrazine, and pyrazine, also showed ROAVs above 1. These compounds therefore contributed substantially to the overall aroma profile. These pyrazine compounds are typically associated with roasted, nutty, and toasted sensory attributes. The highest types and levels of pyrazine and furan compounds were detected in walnut samples, which may be due to phenomena caused by degradation of proteins and amino acids during microwave radiation [20].

In addition to pyrazines, 2-pentylfuran was identified as the only furan compound with ROAV > 1, contributing roasted and nutty notes to the walnut aroma. Several alcohols, including (*E*)-2-penten-1-ol, 2-furanmethanol, 1-pentanol, and 1-octen-3-ol, were also detected as aroma-active compounds, mainly contributing green and fatty notes. Furthermore, (*E*)-2-octenal and 2-hexenal, both unsaturated aldehydes, exhibited ROAVs greater than 1 and are known to impart green, almond-like, and fruity aromas. Aldehydes are predominantly generated through lipid oxidation and, to a lesser extent, amino acid degradation and Strecker reactions [22].

Only one ketone, 3-octen-2-one, was identified among the key aroma compounds, contributing earthy and woody notes. Ketones in roasted nut products can originate from multiple pathways, including lipid oxidation, Maillard reactions, and amino acid degradation. Straight-chain methyl ketones are generally formed through β-oxidation or the oxidative cleavage of free fatty acids. In contrast, branched or unsaturated ketones are more closely associated with amino acid degradation. They can also arise from secondary lipid oxidation processes [23].

Although the types of aroma-active compounds were generally consistent among the five walnut cultivars, differences in ROAVs were observed for several key compounds, indicating cultivar-dependent variations in their aroma contributions. Overall, the highest ROAVs were associated with pyrazines and furans, suggesting that Maillard reaction-derived heterocyclic compounds play a dominant role in the formation of walnut aroma under microwave treatment.

### 3.3. Correlation Between Amino Acid Composition and Key Aroma Compounds

Proteins, fatty acids, and sugars are recognized as the main precursors of walnut aroma. Among them, amino acids play a critical role in flavor development, as they actively participate in the Maillard reaction and Strecker degradation, leading to the formation of key aroma compounds such as pyrazines, furans, and other heterocyclic compounds. In particular, microwave heating has been reported to accelerate these reactions in walnuts, thereby enhancing the generation of roasted and nutty aroma compounds through the combined effects of the Maillard reaction, Strecker degradation, and caramelization [24].

Despite extensive studies on volatile flavor compounds formed during walnut processing, the direct relationship between amino acid composition and aroma formation in walnuts remains largely unexplored. To address this knowledge gap, the present study systematically investigated the amino acid profiles of walnuts processed under microwave treatment, aiming to elucidate their potential contributions to flavor development.

Accordingly, the amino acid compositions of five walnut samples were analyzed in this study, and the results are presented in Table 4. A total of 16 amino acids were detected, including alanine, serine, leucine, aspartic acid, isoleucine, glycine, arginine, histidine, valine, and proline, among others. The five walnut cultivars exhibited broadly similar free amino acid profiles, with glutamic acid, arginine, aspartic acid, leucine, and glycine being the predominant amino acids across all samples. This agreed with the study of Mao [25]. Despite this general similarity, significant cultivar-dependent differences were observed in the absolute concentrations of most amino acids (*p* < 0.05). Among the cultivars, XX2 consistently showed the highest levels of most amino acids, whereas X2 generally exhibited the lowest concentrations. MY1 and Z343 displayed intermediate amino acid contents, while cultivar 185 showed relatively lower levels for several amino acids, particularly sulfur-containing methionine. These results indicate that although the overall amino acid composition pattern was conserved among cultivars, the abundance of individual amino acids varied markedly, reflecting clear varietal effects.

**Figure 2:**
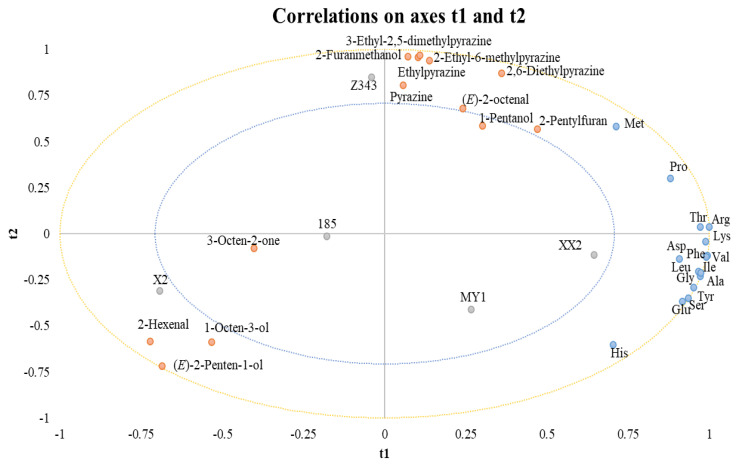
Correlation loadings plots (based on t1 and t2) between amino acid (X), odor active compounds (Y) and sample name (Active) for walnut kernels under microwaved.

Partial least squares regression (PLSR) was applied to explore the relationships between amino acids and 13 key aroma compounds, which were selected based on their ROAVs greater than 1 (Figure 2). In the PLSR loading plot, amino acids were defined as X variables, while odor-active compounds were defined as Y variables. The first two latent components explained 95.3% of the variance in X and 72.2% of the variance in Y, indicating a satisfactory model fit.

All the aroma compounds and amino acids appeared between the two ellipses, indicating that they could be effectively explained by the PLSR model [26]. Several pyrazines and furan derivatives, including 3-ethyl-2,5-dimethylpyrazine, 2-ethyl-6-methylpyrazine, ethylpyrazine, 2,6-diethylpyrazine, pyrazine, 2-pentylfuran, and 2-furanmethanol, exhibited high loadings along the second latent component (t2). These compounds were strongly associated with methionine, indicating that sulfur-containing amino acids may play a key role in promoting the formation of roasted and nutty aroma compounds during microwave treatment.

In addition, the walnut of X2 was correlated with 2-hexenal; 1-octen-3-ol; and (*E*)-2-penten-1-ol and positively correlated with the positive semi-axis of t2. However, the walnuts of XX2 and MY1 clustered in the positive semi-axis region of t1 and may not be associated with any considerable aroma compounds.

Although 1-pentanol and 3-octen-2-one exhibited ROAVs greater than 1 and were therefore included as aroma compounds, their proximity to the origin in the PLSR loading plot suggests a limited contribution to the discrimination of aroma profiles among cultivars. Overall, the PLSR analysis suggests that amino acid composition, particularly methionine, is associated with the generation of key aroma compounds in microwaved walnuts.

### 3.4. The Key Role of Met in Pyrazine Aroma Formation in Microwaved Walnuts

To investigate the differential contribution of amino acids to volatile formation under microwave heating, three independent model reaction systems (Met–glucose, Pro–glucose, and Glu–glucose) were prepared and analyzed in parallel. To improve the accuracy of sample classification and establish a reliable discriminant model, the aroma substances produced by the reaction of different amino acids and glucose were analyzed using the orthogonal partial least squares discriminant analysis (OPLS-DA) method, which is considered an effective method for sample classification and the establishment of discriminant models [27].

**Figure 3:**
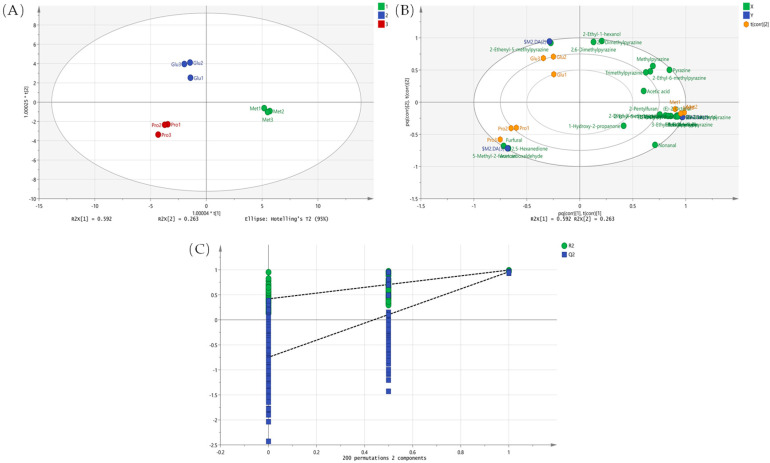
(**A**) OPLS-DA score plot of Glu, Pro, and Met amino acids from walnut samples after oil bath heating using aroma compounds as variables; (**B**) Biplot illustrating the relationship between variables and model components; (**C**) Cross-validation results of the OPLS-DA model.

The OPLS-DA model revealed that the aromatic compounds produced by the three amino acids did not overlap, as illustrated by the score plot (Figure 3A). This indicated strong differentiation, with the model achieving R2X = 0.965, R2Y = 0.979, and Q2 = 0.948. The R2 and Q2 values were close to 1, indicating that the model had good explanatory and predictive properties and a high predictive ability in explaining the variance (R2Y) and cross-validation (Q2). Although OPLS-DA is effective for differentiating samples, it can sometimes lead to overfitting. After 200 substitution tests (Figure 3C), the intersection of the Q2 regression line with the vertical axis was less than zero, indicating no overfitting and validating the robustness of the model. These results are useful for analyzing the generation of aroma compounds from different amino acids.

According to the OPLS-DA model scoring (Figure 3A), Glu is located in the second quadrant and Pro in the third quadrant; however, Met is located in the fourth quadrant. The clear separation between the samples suggests considerable differences in the aroma compounds produced by the three amino acids and glucose, particularly in the production of heterocyclic compounds. Specifically, several pyrazine compounds detected in this study, including 2-ethyl-6-methylpyrazine and 2,6-diethylpyrazine, were strongly associated with the Met–glucose reaction system. The biplot (Figure 3B) shows that Met is closely associated with a cluster of pyrazine compounds that were experimentally identified in the volatile profile of microwave-treated walnuts, whereas Pro was more closely associated with alcohols. Although Glu is linked to certain pyrazines, these associations are weaker and less abundant. This result is consistent with those of previous PLSR analyses. In the PLSR model, Met showed notable positive correlations with several key pyrazines, including 2-ethyl-6-methylpyrazine and 2, 6-diethylpyrazine. This further demonstrated the non-negligible role of Met in the aroma formation of walnuts after microwave treatment. Moreover, this suggests that Met not only showed a strong correlation with pyrazines in the OPLS-DA analysis but also had a key influence on the generation of these aroma compounds in the PLSR analysis.

Overall, the strong correlation between Met and pyrazines during aroma production after the microwave treatment of walnuts suggests that they play an important regulatory role in aroma compound production. However, this effect should be understood as part of a broader reaction network, in which factors such as sugar availability, reaction temperature, and pH may also influence pyrazine formation [28]. This conclusion was supported by both OPLS-DA and PLSR analyses, which further confirmed the position of Met as a key factor in the regulation of pyrazine aromatic substance production.

To explain the formation of pyrazines detected in this study, Yaylayan proposed a reaction mechanism for d-glucose and l-Met production. This reaction mostly occurs between glucose and Met to form Amadori compounds [29]. These compounds are then combined with methionine to form the following two dicarbonyl compounds: 1-deoxyglucosone (1-DG) and 3-deoxyglucosone (3-DG). These intermediates are crucial in the Maillard reaction. Amadori compounds, 1-DG, and 3-DG undergo C3 cleavage via carbonyl migration and reverse hydroxyl aldehyde condensation reactions, thereby yielding intact three-carbon fractions, such as pyruvic alcohols, glyceraldehydes, and glutaraldehydes. All three intermediates generate R-aminocarbonyl compounds via the Strecker reaction, which ultimately produces pyrazine compounds.

Pyrazines are usually formed by the condensation of two α-amino ketone molecules via Strecker degradation of amino acids. This pathway is consistent with the detection of alkyl-substituted pyrazines in the present study, The pH level during this reaction markedly affects the amount of pyrazine formed. Hu observed an unusual pattern for the neutral amino acid cysteine (Cys) [30]. Pyrazine production increases substantially under neutral and slightly alkaline conditions, and walnuts are an alkaline food. Thus, the same sulfur-containing amino acid (Met) showed high pyrazine production in walnuts. Furthermore, pH levels affect pyrazine formation, with low pH levels inhibiting its production owing to the protonation of amino groups in amino acids and peptides.

Considering oxygenated heterocyclic compounds such as furans, sulfur-containing amino acids, such as Cys and Met, are more prone to furan formation. At high temperatures, Met undergoes decarboxylation and deamination to produce methional, which is an important intermediate in the formation of volatile sulfur-containing compounds. Moreover, methionine participates in Strecker degradation by reacting with dicarbonyl compounds, typically generated in the Maillard reaction, to produce Strecker aldehydes, which are then cyclized to form furans. This chain of reactions underscores the multifaceted role of Met in both pyrazine and furan formation, making it a pivotal amino acid in the formation of selected heterocyclic aroma compounds in microwaved walnuts. [31].

## 4. Conclusions

Upon uniform microwave heating of five different walnut varieties respectively, their aroma compounds and concentrations were comparatively analyzed by GC-MS. Based on ROAV analysis, 13 key aroma compounds associated with microwave treatment were identified. The amino acid profiles of these walnuts were assessed, and the results showed correlations between amino acid composition and aroma components. In particular, Met showed a strong association with heterocyclic compounds. A subsequent model reaction experiment further analyzed the effects of heating Met, Pro, Glu, and glucose, supporting the involvement of Met in the formation of heterocyclic aroma compounds under heating conditions. These findings provide insights into the relationship between amino acid composition and aroma formation in microwave-treated walnuts and may contribute to a better understanding of flavor development in walnuts and plant-based protein systems.

## Figures and Tables

**Table 1:** Information on walnut samples used in this study.

Number	Variety	Region
185	185	Aksu
XX2	Xin Xin 2	Aksu
Z343	Zha 343	Aksu
MY1	Mo Yu Thin-Shell No. 1	Hotan
X2	Xin 2	Aksu

**Table 2:** Concentrations of aroma compounds in walnut kernels.

Number Terpenes	LRI (Reference)	LRI (Calculated)	Compound	Concentration (μg/kg) 185 *	Z343 *	X2 *	XX2 *	MY1 *
**1**	1488	1520.6	Pyrrole	30.7 ± 14.25 ^b^	56.25 ± 13.68 ^a^	18.78 ± 3.23 ^bc^	30.51 ± 2.68 ^b^	14.39 ± 6.06 ^c^
**2**	1409	1415.3	Trimethylpyrazine	166.45 ± 73.26 ^c^	582.16 ± 93.54 ^a^	127.11 ± 5.6 ^c^	289.97 ± 18.04 ^b^	93.42 ± 40.55 ^c^
**3**	1275	1285.5	Methylpyrazine	896.87 ± 418.27 ^b^	1466.09 ± 340.93 ^a^	514.35 ± 90.26 ^c^	983.03 ± 90.65 ^b^	306.06 ± 132.43 ^c^
**4**	1350	1343.8	Ethylpyrazine	117.9 ± 53.56 ^bc^	228.54 ± 50.32 ^a^	89.19 ± 11.41 ^cd^	142.54 ± 15.54 ^b^	51.87 ± 22.44 ^d^
**5**	1445	1451.8	3-Ethyl-2,5-dimethyl-pyrazine	36.5 ± 14.56 ^c^	163.58 ± 26.7 ^a^	31.29 ± 1.74 ^c^	64.42 ± 4.34 ^b^	21.93 ± 9.29 ^c^
**6**	1403	1398.4	2-Ethyl-6-methylpyrazine	193.47 ± 84.25 ^c^	666.17 ± 127.45 ^a^	214.88 ± 12.79 ^c^	350.03 ± 16.96 ^b^	156.24 ± 63.73 ^c^
**7**	1347	1335.3	2,6-Dimethylpyrazine	241.12 ± 110.43 ^b^	699.9 ± 156.91 ^a^	126.11 ± 11.51 ^c^	305.3 ± 34.99 ^b^	71.83 ± 32.34 ^c^
**8**	1437	1443.5	2,6-Diethylpyrazine	85.86 ± 36.71 ^c^	285.9 ± 32.95 ^a^	74.82 ± 0.63 ^c^	198.52 ± 1.07 ^b^	72.26 ± 29.26 ^c^
**9**	1339	1328.7	2,5-Dimethylpyrazine	689.93 ± 313.48 ^c^	1915.65 ± 412.57 ^a^	713.19 ± 95.02 ^c^	1202.12 ± 32.91 ^b^	529.25 ± 218.88 ^c^
**10**	1353	1356.5	2,3-Dimethylpyrazine	83.1 ± 38.54 ^b^	146.14 ± 27.95 ^a^	45.33 ± 3.83 ^c^	92.07 ± 9.18 ^b^	24.22 ± 9.98 ^c^
**11**	1214	1225.6	Pyrazine	51.56 ± 23.36 ^ab^	55.3 ± 11.15 ^a^	17.12 ± 4.25 ^c^	36.26 ± 6.18 ^b^	8.36 ± 3.38 ^c^
**12**	1638	1652.9	Acetylpyrazine	4.09 ± 1.39 ^b^	9.3 ± 0.71 ^a^	2.02 ± 0.22 ^c^	4.04 ± 0.98 ^b^	0.73 ± 0.11 ^d^
**13**	1244	1234.2	2-Pentylfuran	47.91 ± 24.98 ^c^	265.03 ± 48.78 ^a^	125.52 ± 3.99 ^b^	268.08 ± 20.4 ^a^	101.51 ± 31.51 ^b^
**14**	-	1751.4	Pentanoic acid	4.75 ± 2.78 ^d^	97.38 ± 18.93 ^a^	22.8 ± 2.07 ^c^	73.75 ± 14.35 ^b^	37.4 ± 15.04 ^c^
**15**	2042	1861.3	Hexanoic acid	31.68 ± 18.88 ^e^	1026.01 ± 150.02 ^a^	192.78 ± 40.58 ^d^	726.88 ± 76.69 ^b^	369.16 ± 130.85 ^c^
**16**	1473	1472.7	Acetic acid	245.07 ± 102.74 ^b^	361.81 ± 35.25 ^a^	188.88 ± 11.3 ^bc^	212.63 ± 41.79 ^b^	117.51 ± 45.95 ^c^
**17**	1289	1291.6	Octanal	2.53 ± 1.15 ^d^	17.49 ± 2.74 ^a^	10.54 ± 0.06 ^c^	8.11 ± 0.75 ^c^	13.4 ± 3.22 ^b^
**18**	-	1475.3	Furfural	759.87 ± 357.43 ^ab^	980.69 ± 74.67 ^a^	541.16 ± 101.25 ^b^	727.01 ± 94.73 ^b^	200.29 ± 78.14 ^c^
**19**	-	1539.8	Benzaldehyde	32.67 ± 17.53 ^b^	19.25 ± 2.35 ^b^	50.01 ± 9.12 ^a^	30.43 ± 0.51 ^b^	30.71 ± 9.86 ^b^
**20**	1437	1442.2	(*E*)-2-Octenal	12.27 ± 5.93 ^d^	55.88 ± 9.77 ^a^	29.55 ± 0.84 ^c^	38.05 ± 0.2 ^b^	29.91 ± 7.4 ^c^
**21**	1234	1225.2	2-Hexenal	32.43 ± 16.38 ^b^	20.88 ± 5.09 ^b^	67.2 ± 16.87 ^a^	28.22 ± 0.32 ^b^	34.31 ± 11.89 ^b^
**22**	1335	1333.2	(*Z*)-2-Heptenal	152.69 ± 77.24 ^bc^	96.41 ± 15.86 ^c^	269.1 ± 50.66 ^a^	143.09 ± 2.63 ^bc^	189.93 ± 59.67 ^b^
**23**	1655	1663	(*E*)-2-Decenal	22.47 ± 10.59 ^bc^	54.62 ± 2.4 ^a^	15.14 ± 1.42 ^c^	28.53 ± 9.42 ^b^	0.81 ± 0.11 ^d^
**24**	1507	1514.5	(*E,E*)-2,4-Heptadienal	214.8 ± 102.9 ^b^	101.43 ± 10.84 ^c^	391.8 ± 32.66 ^a^	178.16 ± 2.25 ^b^	198.53 ± 54.73 ^b^
**25**	1862	1829.5	(*E,E*)-2,4-Decadienal	20.77 ± 8.18 ^d^	16.2 ± 0.89 ^d^	52.93 ± 5.64 ^a^	45.58 ± 1.09 ^b^	29.71 ± 4.18 ^c^
**26**	1603	1609.2	(*E*)-2-Octen-1-ol	1.65 ± 0.87 ^d^	4.02 ± 0.41 ^b^	5.67 ± 0.45 ^a^	3.14 ± 0.12 ^c^	4.18 ± 0.79 ^b^
**27**	1328	1316.2	(*Z*)-2-Penten-1-ol	14.11 ± 6.62 ^b^	5.1 ± 0.84 ^c^	24.76 ± 6.65 ^a^	8.88 ± 2.15 ^bc^	14.84 ± 6.22 ^b^
**28**	1679	1665.5	2-Furanmethanol	108.91 ± 53.55 ^bc^	269.92 ± 10.66 ^a^	73.27 ± 6.6 ^c^	140.57 ± 45.45 ^b^	14.49 ± 6.47 ^d^
**29**	1260	1252	1-Pentanol	55.21 ± 26.17 ^c^	172.78 ± 38.5 ^a^	98.5 ± 30.81 ^b^	119.82 ± 5.74 ^b^	125.7 ± 45.22 ^b^
**30**	1452	1455	1-Octen-3-ol	154.21 ± 76.43 ^c^	164.24 ± 24.4 ^bc^	321.03 ± 57.71 ^a^	187.53 ± 2.09 ^bc^	235.21 ± 71.52 ^b^
**31**	1361	1350.6	1-Hexanol	126.1 ± 66.11 ^c^	484.19 ± 101.74 ^a^	181.78 ± 48.62 ^bc^	106.64 ± 3.78 ^c^	226.3 ± 77.06 ^b^
**32**	1441	1419.5	3-Octen-2-one	10.98 ± 5.83 ^b^	23.36 ± 4.65 ^a^	28.41 ± 2.08 ^a^	15.92 ± 1.26 ^b^	24.84 ± 6.78 ^a^

* 185, 185 variety; Z343, Zha 343; X2; Xin 2 variety; XX2, Xin Xin 2 variety; MY1, Mo-Yu Thin-Shell No. 1 variety. ^a,b,c,d,e^ Means within the same column followed by different lowercase letters are significantly different (*p*< 0.05); those sharing the same letter are not significantly different (*p* > 0.05).

**Table 3:** Odor description and ROAV of volatile compounds in different walnut varieties.

Class	Compound	Odor Description	Odor Threshold (μg/kg)	ROAV 185 *	Z343 *	X2 *	XX2 *	MY1 *
Heterocyclic	Pyrrole	nutty, roasted	10	0.0079	0.0042	0.0044	0.0039	0.0046
Compounds	Trimethylpyrazine	earthy, nutty	6.55	0.0657	0.0667	0.0452	0.0561	0.0456
	Methylpyrazine	nutty, cocoa-like	27	0.0858	0.0408	0.0443	0.0462	0.0363
	Ethylpyrazine	nutty, roasted	0.25	1.2188	0.6861	0.8301	0.7231	0.6639
	3-Ethyl-2,5-dimethylpyrazine	nutty, roasted	0.084	1.1230	1.4616	0.8668	0.9726	0.8353
	2-Ethyl-6-methyl pyrazine	roasted, nutty	0.005	100.0000	100.0000	100.0000	88.7866	100.0000
	2,6-DimethylPyrazine	nutty, cocoa-like	130	0.0048	0.0040	0.0023	0.0030	0.0018
	2,6-Diethyplyrazine	roasted, nutty	0.05	4.4379	4.2917	3.4819	5.0355	4.6249
	2,5-Dimethylpyrazine	nutty, roasted	120	0.0149	0.0120	0.0138	0.0127	0.0141
	2,3-Dimethylpyrazine	nutty, roasted	880	0.0002	0.0001	0.0001	0.0001	0.0001
	Pyrazine	nutty, roasted	0.02	6.6619	2.0754	1.9919	2.2991	1.3375
	Acetylpyrazine	nutty, cocoa-like	62	0.0001	0.0005	0.0004	0.0000	0.0000
	2-Pentyl Furan, -	fruity, nutty	5.8	1.9220	3.8610	7.2159	100.0000	95.5468
Alcohols	1-Octen-3-ol	mushroom, earthy	0.153	0.9326	0.8476	1.4980	1.5545	4.9196
	1-Hexanol	green, floral	1	0.3985	0.1233	0.7470	0.1353	0.7242
Acids	Pentanoic acid	cheesy, sour	138	0.0002	0.0017	0.0010	0.0002	0.0003
	Hexanoic acid	sour, fatty	460	0.0065	0.0025	0.0042	0.0031	0.0039
	Acetic acid	sour, vinegar-like	350	0.0213	0.0343	0.0504	0.0465	0.0648
Aldehydes	Octanal	fatty, citrus, honey	0.0034	0.0008	0.0001	0.0012	0.0001	0.0004
	Furfural	caramel, cinnamon, and almond	300	0.0002	0.0001	0.0001	0.0149	0.0103
	Benzaldehyde	bitter almond	100	0.0018	0.0008	0.0013	0.0001	0.0003
	(E)-2-Octenal	green, fatty	3	4.2360	2.6169	9.8684	7.2033	14.2881
	2-Hexenal	fruity, green, vegetable-like	0.85	3.6476	0.3824	5.7611	3.5796	10.9789
	(Z)-2-Heptenal	fruity, fatty	1.93	0.0106	0.0140	0.0229	0.0605	0.2026
	(E)-2-Decenal	citrus, fatty	0.03	0.0568	0.0402	0.1760	0.4825	0.0346
	(E,E)-2,4-Heptadienal	green, fatty	0.1	0.0986	0.0184	0.1840	0.2658	0.7474
	(E,E)-2,4-Decadienal	citrus, fatty	0.07	0.2045	0.0375	0.3244	0.0300	0.0493
Ketones	3-Octen-2-one	mushroom, earthy	0.034	**9.5848**	**10.6886**	**12.4408**	0.5938	**2.3377**
Other	D-Limonene	citrus, sweet	0.045	0.2681	0.0995	0.7356	0.0862	0.9753
Alcohols	(E)-2-Octen-1-ol	green, mushroom	0.075	0.0001	0.0000	0.0000	0.0000	0.0000
	(E)-2-Penten-1-ol	green, floral	4500.5	**1.9358**	**1.3666**	**1.1747**	0.3755	**1.5831**
	2-Furanmethanol	sweet, caramel	0.0067	**5.5513**	0.7613	**9.1170**	**1.7828**	0.4636
	1-Pentanol	fusel-like	0.01	0.7670	0.1737	**1.7596**	**2.1710**	**5.7466**

* Note: a, odor threshold values were taken from <Odour Thresholds> and refer to values reported in a plant oil matrix. b, ROAV, relative odor activity value; 185, 185 variety; Z343, Zha 343; X2; Xin 2 variety; XX2, Xin Xin 2 variety; MY1, Mo-Yu Thin-Shell No. 1 variety.

**Table 4:** Amino acid profiles of different walnut variety samples (g/100 g).

Variety	Alanine	Serine	Leucine	Aspartic Acid	Isoleucine	Glycine	Arginine	Histidine	Valine	Proline	Threonine	Phenylalanine	Methionine	Glutamic Acid	Lysine	Tyrosine
185	0.6916 ± 0.0245 ^b^	0.8297 ± 0.0382 ^bc^	1.1211 ± 0.0575 ^b^	1.4591 ± 0.0714 ^b^	0.6218 ± 0.0397 ^bc^	0.8385 ± 0.0490 ^bc^	2.4347 ± 0.1562 ^bc^	0.3917 ± 0.0244 ^bc^	0.7086 ± 0.0502 ^bc^	0.5031 ± 0.0034 ^b^	0.5361 ± 0.0243 ^b^	0.7081 ± 0.0406 ^bc^	0.0488 ± 0.0040 ^d^	3.4849 ± 0.1873 ^c^	0.4510 ± 0.0267 ^bc^	0.5312 ± 0.0221 ^bc^
Z343	0.7021 ± 0.0080 ^b^	0.8034 ± 0.0062 ^b^	1.1177 ± 0.0064 ^b^	1.4975 ± 0.0143 ^b^	0.6201 ± 0.0086 ^bc^	0.8218 ± 0.0061 ^b^	2.4836 ± 0.0211 ^b^	0.3894 ± 0.0059 ^abc^	0.7116 ± 0.0057 ^bc^	0.5660 ± 0.0054 ^a^	0.5346 ± 0.0051 ^b^	0.7169 ± 0.0007 ^b^	0.1451 ± 0.0012 ^c^	3.3587 ± 0.0743 ^bc^	0.4479 ± 0.0086 ^ab^	0.5145 ± 0.0084 ^b^
X2	0.6716 ± 0.0220 ^b^	0.7788 ± 0.0031 ^c^	1.0593 ± 0.0064 ^b^	1.4504 ± 0.0240 ^b^	0.5720 ± 0.0029 ^c^	0.7671 ± 0.0074 ^c^	2.2701 ± 0.0041 ^c^	0.4026 ± 0.0007 ^c^	0.6661 ± 0.0001 ^c^	0.3508 ± 0.0040 ^c^	0.4557 ± 0.0004 ^c^	0.6616 ± 0.0045 ^c^	0.0340 ± 0.0020 ^e^	3.3064 ± 0.0006 ^c^	0.4137 ± 0.0002 ^c^	0.4930 ± 0.0096 ^c^
XX2	0.7684 ± 0.0054 ^a^	0.9125 ± 0.0057 ^a^	1.2432 ± 0.0017 ^a^	1.6823 ± 0.0565 ^a^	0.6908 ± 0.0095 ^a^	0.9305 ± 0.0059 ^a^	2.6941 ± 0.0218 ^a^	0.4310 ± 0.0077 ^a^	0.7846 ± 0.0054 ^a^	0.5771 ± 0.0148 ^a^	0.5908 ± 0.0024 ^a^	0.7970 ± 0.0034 ^a^	0.1588 ± 0.0032 ^a^	3.8402 ± 0.0460 ^a^	0.4869 ± 0.0118 ^a^	0.5901 ± 0.0062 ^a^
MY1	0.7475 ± 0.0004 ^a^	0.8855 ± 0.0153 ^a^	1.2105 ± 0.0068 ^a^	1.5470 ± 0.0055 ^b^	0.6636 ± 0.0062 ^ab^	0.8989 ± 0.0027 ^a^	2.5601 ± 0.0205 ^ab^	0.4222 ± 0.0016 ^ab^	0.7521 ± 0.0083 ^ab^	0.5842 ± 0.0156 ^a^	0.5694 ± 0.0068 ^a^	0.7692 ± 0.0104 ^a^	0.0657 ± 0.0039 ^b^	3.6528 ± 0.0364 ^ab^	0.4684 ± 0.0063 ^ab^	0.5646 ± 0.0082 ^a^

^a,b,c,d,e^ Means within the same column followed by different lowercase letters are significantly different (*p* < 0.05); those sharing the same letter are not significantly different (*p* > 0.05).

## Data Availability

The original contributions presented in the study are included in the article/supplementary material, further inquiries can be directed to the corresponding author.

## Supplementary Materials

The following supporting information can be downloaded at: https://www.mdpi.com/article/10.3390/foods15040719/s1, Figure S1. Heatmap of volatile compounds in untreated walnut samples from different cultivars. Figure S2. Correlation heatmap between free amino acids and key volatile compounds in microwaved walnut samples.
