# Supplementary material for: The Role of Methionine in the Formation of Key Aroma Compounds in Microwaved Walnuts
Source: Foods. 2026 Feb 15;15(4):719. doi: 10.3390/foods15040719 (PMC12940861; doi:10.3390/foods15040719)
Supplement: Supplementary file 1 [file foods-15-00719-s001.zip › foods-4128623-supplementary.pdf]

foods

Supplementary Material

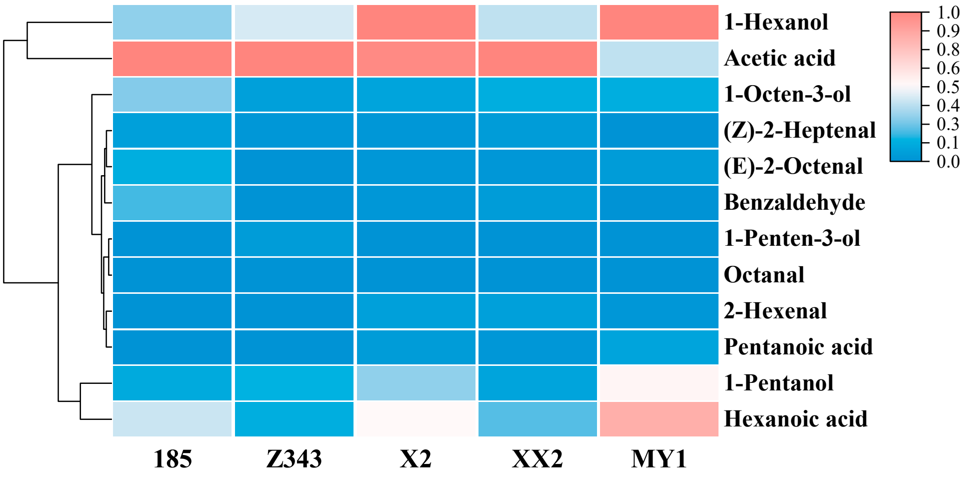

Figure S1. Heatmap of volatile compounds in untreated walnut samples from different cultivars

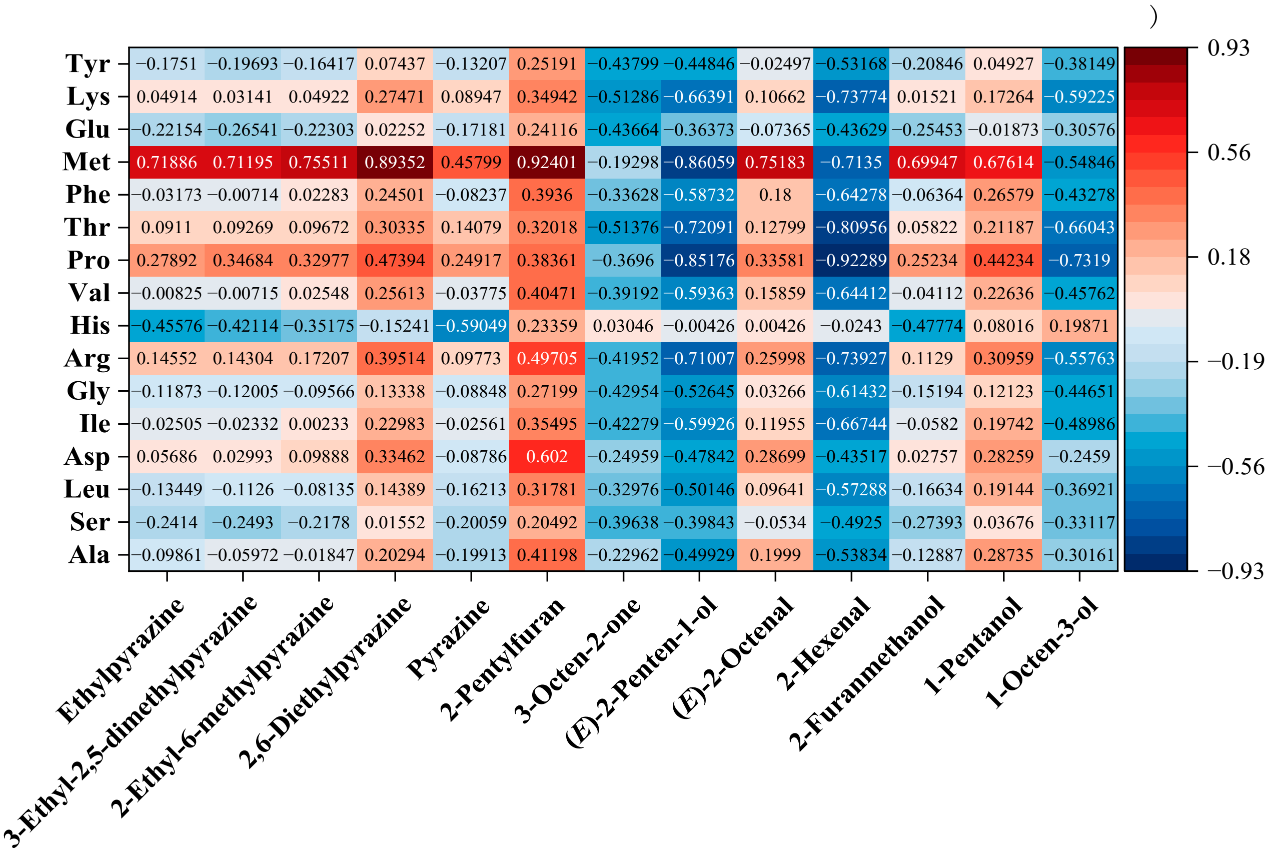

Figure S2. Correlation heatmap between free amino acids and key volatile compounds in microwaved walnut samples
